# Supplementary material for: Prothrombin Time-International Normalized Ratio Predicts the Outcome of Atrial Fibrillation Patients Taking Rivaroxaban
Source: Biomedicines. 2022 Dec 10;10(12):3210. doi: 10.3390/biomedicines10123210 (PMC9775588; doi:10.3390/biomedicines10123210)
Supplement: Supplementary file 1 [file biomedicines-10-03210-s001.zip › biomedicines-2019562-supplementary.pdf]

Supplemental Material

Supplemental Table S1

Risk of IS/SE and major bleeding for patients treated with rivaroxaban in different INR categories

		**Crude HR**		**Adjusted HR**	
**IS/SE**					
	**Event Rate/100 Person-Years (95% CI)**	**HR (95% CI)**	***P* value**	**HR (95% CI)***	***P* value**
**Rivaroxaban INR < 1.1**	3.27 (2.52 – 4.03)	**Ref**	**Ref**	**Ref**	**Ref**
**Rivaroxaban INR 1.1~1.2**	3.10 (2.49 – 3.71)	0.95 (0.70-1.29)	0.74	0.95 (0.70-1.29)	0.74
**Rivaroxaban INR 1.3~1.4**	2.58 (1.64 – 3.52)	0.80 (0.52-1.22)	0.30	0.81 (0.52-1.24)	0.33
**Rivaroxaban INR ≥ 1.5**	1.82 (1.18 – 2.13)	**0.57 (0.37-0.86)**	**0.01**	**0.57 (0.37-0.87)**	**0.01**
**Major bleeding**					
	**Event Rate/100 Person-Years (95% CI)**	**HR (95% CI)**	***P* value**	**HR (95% CI)†**	***P* Value**
**Rivaroxaban INR < 1.1**	2.70 (2.02 – 3.38)	**Ref**	**Ref**	**Ref**	**Ref**
**Rivaroxaban INR 1.1~1.2**	2.47 (1.94 – 3.00)	0.92 (0.66-1.27)	0.60	0.88 (0.63-1.23)	0.44
**Rivaroxaban INR 1.3~1.4**	2.36 (1.47 – 3.25)	0.88 (0.56-1.38)	0.56	0.90 (0.57-1.41)	0.63
**Rivaroxaban INR ≥ 1.5**	2.17 (1.47 – 2.86)	0.81 (0.54-1.21)	0.30	0.85 (0.56-1.29)	0.44

CI = confidential interval; eGFR = estimated glomerular filtration rate; HR = hazard ratio; INR = international normalized ratio; IS/SE = Ischemic stroke or systemic embolism; NVAF = nonvalvular atrial fibrillation.

*Adjusted for age, female gender, CHA_2DS_2-VASc score and eGFR

†Adjusted for age, female gender, HASE-BLED score and eGFR

Supplemental Table S2

Risk of IS/SE and major bleeding for patients treated with on-label dosing of rivaroxaban in different INR categories

		**Crude HR**		**Adjusted HR**	
**IS/SE**					
	**Event Rate/100 Person-Years (95% CI)**	**HR (95% CI)**	***P* value**	**HR (95% CI)***	***P* value**
**Rivaroxaban INR < 1.1**	3.18 (2.27 – 4.09)	**Ref**	**Ref**	**Ref**	**Ref**
**Rivaroxaban INR 1.1~1.2**	2.56 (1.90 – 3.21)	0.81 (0.55-1.19)	0.27	0.81 (0.55-1.19)	0.28
**Rivaroxaban INR 1.3~1.4**	2.67 (1.58 – 3.76)	0.84 (0.51-1.39)	0.51	0.84 (0.51-1.38)	0.48
**Rivaroxaban INR ≧ 1.5**	1.80 (1.06 – 2.53)	**0.57 (0.35-0.94)**	**0.03**	**0.57 (0.35-0.94)**	**0.03**
**Major bleeding**					
	**Event Rate/100 Person-Years (95% CI)**	**HR (95% CI)**	***P* value**	**HR (95% CI)†**	***P* Value**
**Rivaroxaban INR < 1.1**	2.92 (2.06 – 3.79)	**Ref**	**Ref**	**Ref**	**Ref**
**Rivaroxaban INR 1.1~1.2**	2.46 (1.83 – 3.09)	0.85 (0.57-1.25)	0.41	0.83 (0.56-1.24)	0.36
**Rivaroxaban INR 1.3~1.4**	2.50 (1.45 – 3.54)	0.86 (0.51-1.43)	0.56	0.87 (0.52-1.46)	0.60
**Rivaroxaban INR ≧ 1.5**	2.19 (1.38 – 3.00)	0.75 (0.47-1.20)	0.23	0.77 (0.48-1.24)	0.29

CI = confidential interval; eGFR = estimated glomerular filtration rate; HR = hazard ratio; INR = international normalized ratio; IS/SE = Ischemic stroke or systemic embolism; NVAF = nonvalvular atrial fibrillation.

*Adjusted for age, female gender, CHA_2DS_2-VASc score and eGFR

†Adjusted for age, female gender, HASE-BLED score and eGFR

Supplemental Table S3

Risk of IS/SE and major bleeding for patients treated with dabigatran in different aPTT ratio categories

		**Crude HR**		**Adjusted HR**	
**IS/SE**					
	**Event Rate/100 Person-Years (95% CI)**	**HR (95% CI)**	***P* value**	**HR (95% CI)***	***P* value**
**Dabigatran aPTT ratio < 1.1**	3.76 (2.48 – 5.04)	**Ref**	**Ref**	**Ref**	**Ref**
**Dabigatran aPTT ratio 1.1~1.2**	1.67 (0.73 – 2.62)	**0.49 (0.26-0.92)**	**0.03**	**0.50 (0.26-0.95)**	**0.04**
**Dabigatran aPTT ratio 1.3~1.4**	1.51 (0.50 – 2.51)	**0.40 (0.19-0.84)**	**0.02**	**0.38 (0.18-0.81)**	**0.01**
**Dabigatran aPTT ratio ≥ 1.5**	2.48 (1.23 – 3.74)	0.64 (0.35-1.17)	0.15	0.63 (0.34-1.17)	0.14
**Major bleeding**					
	**Event Rate/100 Person-Years (95% CI)**	**HR (95% CI)**	***P* value**	**HR (95% CI)†**	***P* value**
**Dabigatran aPTT ratio < 1.1**	2.00 (1.06 – 2.94)	**Ref**	**Ref**	**Ref**	**Ref**
**Dabigatran aPTT ratio 1.1~1.2**	0.98 (0.27 – 1.69)	0.45 (0.19-1.06)	0.07	0.50 (0.21-1.18)	0.11
**Dabigatran aPTT ratio 1.3~1.4**	1.08 (0.16 – 1.99)	0.61 (0.27-1.39)	0.24	0.61 (0.27-1.40)	0.24
**Dabigatran aPTT ratio ≥ 1.5**	2.53 (1.23 – 3.83)	1.24 (0.65-2.36)	0.52	1.14 (0.59-2.19)	0.70

CI = confidential interval; eGFR = estimated glomerular filtration rate; HR = hazard ratio; INR = international normalized ratio; IS/SE = Ischemic stroke or systemic embolism; NVAF = nonvalvular atrial fibrillation.

*Adjusted for age, female gender, CHA_2DS_2-VASc score and eGFR

†Adjusted for age, female gender, HASE-BLED score and eGFR
